# Importance of Plasmonic Heating on Visible Light Driven Photocatalysis of Gold Nanoparticle Decorated Zinc Oxide Nanorods

**DOI:** 10.1038/srep26913

**Published:** 2016-05-31

**Authors:** Tanujjal Bora, David Zoepfl, Joydeep Dutta

**Affiliations:** 1 Chair in Nanotechnology, Sultan Qaboos University, PO Box 17, Al Khoud–123, Oman; 2 Institute for Quantum Optics and Quantum Information, Austrian Academy of Sciences, Technikerstraße 21a, A-6020 Innsbruck, Austria; 3 Functional Materials Division, Materials and Nano Physics Department, ICT School, KTH Royal Institute of Technology, SE-164 40, Kista, Stockholm, Sweden

## Abstract

Herein we explore the role of localized plasmonic heat generated by resonantly excited gold (Au) NPs on visible light driven photocatalysis process. Au NPs are deposited on the surface of vertically aligned zinc oxide nanorods (ZnO NRs). The localized heat generated by Au NPs under 532 nm continuous laser excitation (SPR excitation) was experimentally probed using Raman spectroscopy by following the phonon modes of ZnO. Under the resonant excitation the temperature at the surface of the Au-ZnO NRs reaches up to about 300 °C, resulting in almost 6 times higher apparent quantum yield (AQY) for photocatalytic degradation of methylene blue (MB) compared to the bare ZnO NRs. Under solar light irradiation the Au-ZnO NRs demonstrated visible light photocatalytic activity twice that of what was achieved with bare ZnO NRs, while significantly reduced the activation energy required for the photocatalytic reactions allowing the reactions to occur at a faster rate.

Plasmonic photocatalysts composed of noble metal nanoparticles (NPs) deposited on the surface of semiconductor materials have recently shown promising results towards the visible light driven photocatalysis, where metal NPs act as sensitizers for harvesting visible light due to their surface plasmon resonance (SPR) and the metal-semiconductor interface efficiently separates the photo-generated charges^1^,^2^,^3^,^4^. Incorporation of metal NPs, such as gold (Au) and silver (Ag) into the semiconductor photocatalysts brings drastic enhancement in the photocatalytic activity under both ultraviolet (UV) and visible light irradiation, owing to the several advantages offered by the metal-semiconductor composites, such as enhanced visible light harvesting due to the localized SPR effect from the metal NPs, reduced electron-hole (e-h) pair diffusion length, efficient photo-generated charge separation and transfer and localized heating effect from the metal NPs^5^,^6^.

In recent years, several researchers have reported efficient visible light photocatalysis by using Au or Ag NPs deposited on traditional semiconductor photocatalysts like titanium dioxide (TiO_2_) and zinc oxide (ZnO)^5^,^7^,^8^,^9^,^10^. Being wide bandgap semiconductor materials traditional photocatalysts require UV light for activation and thus they can use only about 4% of the solar spectrum for photocatalysis. Although there are number of methods to activate the wide bandgap semiconductors under the visible light, such as doping or sensitization with visible light active organic dyes or other semiconductor quantum dots^11^,^12^,^13^,^14^; these methods have their own limitations like complicated synthesis processes and long term stability issues. Application of noble metal NPs, in this regard can provide improved long term stability compared to the organic dyes and they can be prepared cost effectively using simple synthesis routes. Pincella *et al.*^3^ has reported a wet chemical bottom-up approach to fabricate Au-TiO_2_ plasmonic photocatalysts demonstrating visible light photocatalytic activity against organic dye molecules. In one of our recent works, we have also demonstrated a simple room temperature wet chemical method to deposit Au NPs on supported ZnO NRs and their application for visible light photocatalysis^5^. While majority of the reported literatures attribute the enhanced visible light photocatalytic activity of metal-semiconductor plasmonic photocatalysts to its improved visible light harvesting and efficient photo-generated charge separation within the material^15^; other contributing factors like localized plasmonic heating from the metal nanoparticles that can significantly alter the activation barrier of the heterogeneous photocatalytic process allowing the reactions to proceed at a faster rate has however not been considered^16^.

Direct estimation of localized plasmonic heating is a challenging task, and in this regard several theoretical models have been proposed till date to quantify the SPR-induced temperature rise at the surface of optically excited metal NPs^17^,^18^,^19^. However, recently few attempts have been made to experimentally determine the SPR induced surface temperature changes on metal nanostructures. Nanoscale “thermometers” to measure the local temperatures on optically excited plasmonic NPs surface in different environments have been reported by several groups^20^,^21^. Qiu *et al.*^22^ has developed a chemical-reaction-based molecular thermometer to measure the SPR-induced surface temperature on a nanostructured silver substrate, where they have used surface enhanced Raman spectroscopy (SERS) to quantify the surface temperature. Richardson *et al.*^23^ has introduced another approach using thermooptical spectroscopy based on phase transformation of a matrix, which can not only measure optical response but also thermal response at the surface of the meal nanostructures. Another group has used metal NPs coupled to a semiconductor with a polymer chain, where they have monitored the emission intensity of the semiconductor which is highly dependent on the chain length of the polymer that varies with surrounding temperature induced by the SPR heating^24^. Recently Raman spectroscopy was also applied to quantify the SPR induced surface temperature in Au-ZnO nanocomposite, where the increase in the surface temperature was estimated by monitoring the phonon modes of ZnO^25^.

In this work, we have explored the contribution of localized plasmonic heating from resonantly excited Au NPs in Au-ZnO plasmonic photocatalysts on their visible light photocatalytic activity. The surface temperature of Au NPs decorated ZnO NRs were experimentally determined by following the phonon modes of ZnO using Raman spectroscopy. The effect of the localized heating on the apparent quantum yield (AQY) and activation energy (E_a_) of the Au-ZnO system was also investigated, along with the other major contributing factors such as enhanced visible light harvesting and efficient photo-generated charge separation across the Au-ZnO interface in order to obtain a detailed insight on the visible light driven plasmonic photocatalysis by metal-semiconductor systems.

## Results and Discussion

**Figure 1:**
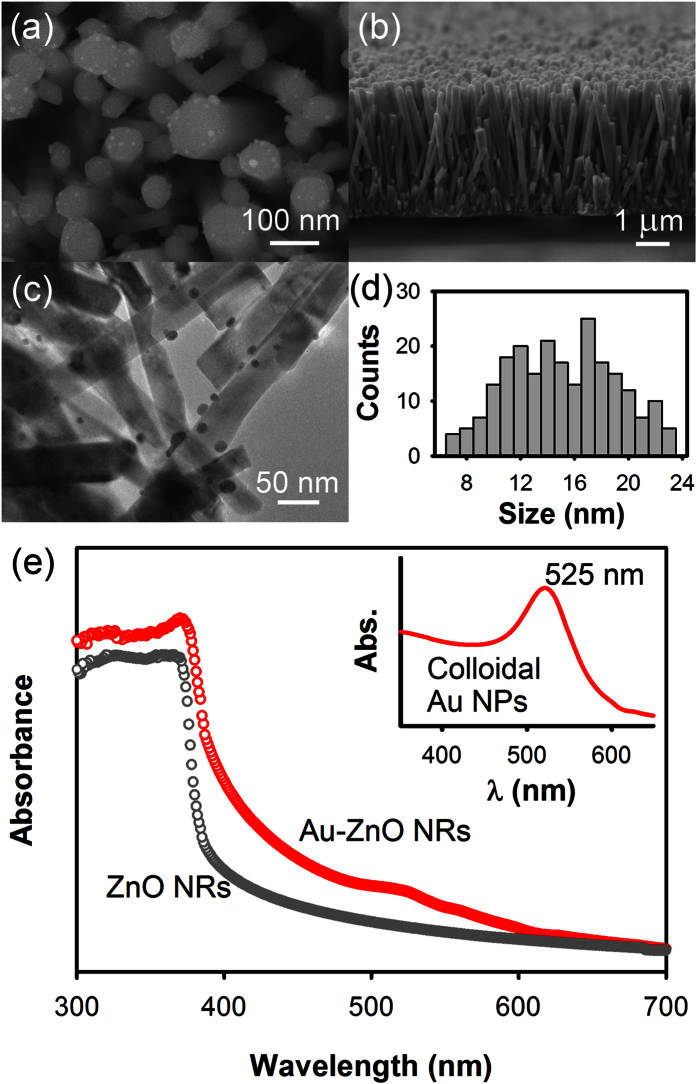
Scanning electron micrographs of Au-ZnO NRs showing (**a**) the top view and (**b**) the cross-sectional view of the NRs. (**c**) A typical TEM micrograph of the Au-ZnO NRs along with (**d**) the size distribution of the Au NPs. Au NPs were deposited *in-situ* on the surface of the ZnO NRs for 10 minutes by photocatalytic reduction of chloroauric acid (HAuCl_4_.3 H_2_O) under UV light irradiation. (**e**) UV/Vis optical absorption spectra of bare ZnO and Au-ZnO NRs. Inset shows the UV/Vis optical absorption spectrum of ~20 nm colloidal Au NPs for comparison with the surface plasmon absorption band peaking at 525 nm.

The scanning electron micrograph of Au-ZnO NRs is shown in Fig. 1(a), where Au NPs deposited on the surface of the ZnO NRs are clearly visible. ZnO NRs showed the characteristic hexagonal shape with diameter ranging from 50–100 nm and length about 4 μm as shown in Fig. 1(b). Figure 1(c) shows a typical TEM micrograph of the Au-ZnO NRs, where *in-situ* deposition of the Au NPs was carried out for 10 minutes. The size distribution of the Au NPs, estimated from multiple TEM micrographs, indicates particle diameter ranging from 7–23 nm, as shown in Fig. 1(d). The wide size distribution obtained for the Au NPs is expected here since the *in-situ* deposition of Au NPs on the surface of the ZnO NRs was carried out without using any capping agents which provides less size controllability. The amount of Au loading on these Au-ZnO NRs was estimated by using energy dispersive X-ray (EDX) technique, which showed Au concentration as 2.6 ± 0.2 wt. % for 10 minutes deposition time. Details of the EDX analysis are given in the supplementary information (Fig. S1), where variation in the Au loading with respect to the deposition time is shown. From this point onwards we will discuss only the results from the Au-ZnO NRs where Au NPs were deposited for 10 minutes, as these samples showed maximum visible light photocatalytic activities. Figure 1(e) shows the optical absorption spectra of bare ZnO and Au-ZnO NRs in the ultraviolet-visible spectral region. The bare ZnO NRs exhibit high absorption band in the UV range (below 370 nm) due to its high extinction coefficients in this region. In case of the Au-ZnO NRs, the spectrum showed two absorption bands, one in the UV region similar to the bare ZnO NRs and another in the visible region centered at around 525 nm. The UV band corresponds to the ZnO NRs and the visible band near 525 nm represents the characteristic surface plasmon absorption band of Au NPs present on the surface of the ZnO NRs, making the Au-ZnO NRs active under both UV and visible light region. For comparison, an optical absorption spectrum of colloidal Au NPs (size ~20 nm) is shown in the inset indicating the characteristic surface plasmon absorption peak of Au NPs at 525 nm. Details of the synthesis and size distribution of the colloidal Au NPs are given in the supplementary information (Fig. S2). Similar results were also reported previously by several groups^26^,^27^,^28^.

**Figure 2:**
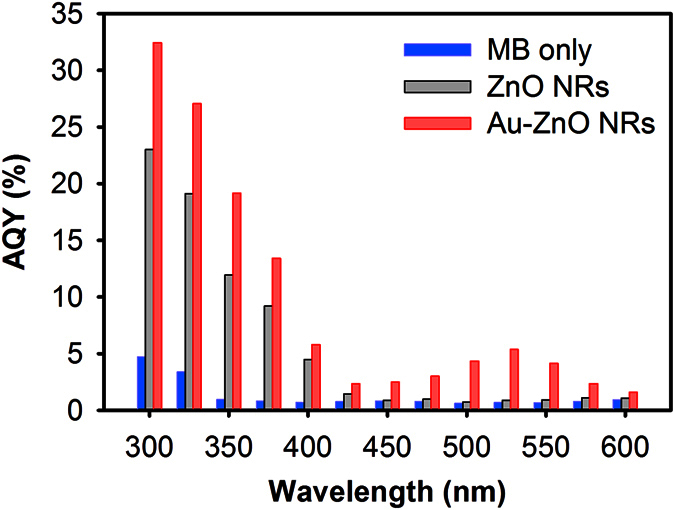
Apparent quantum yield (AQY) estimated for the photocatalytic degradation of MB with bare ZnO NRs, Au-ZnO NRs and without any photocatalysts at different incident monochromatic wavelengths (Exposure time: 30 minutes).

In order to explore the plasmonic effects of Au NPs on the visible light photocatalysis, we have initially studied the photocatalytic degradation of methylene blue (MB) using bare ZnO and Au-ZnO NRs under different monochromatic excitation wavelengths and estimated the apparent quantum yield (AQY) of the photocatalytic process, as shown in Fig. 2. The detailed calculation process of AQY for the photocatalytic processes is given in the Methods section. MB solution in absence of any photocatalyst showed very low AQY values in all excitation wavelengths. In the UV region, both bare ZnO and Au-ZnO NRs exhibited high AQY values, attributed to their high UV absorption bands observed in Fig. 1(c). Gold typically forms Schottky junction with ZnO creating a space charge region in the ZnO side but near the metal-semiconductor interface, which upon photo-excitation, would force the electrons and holes to move in different directions once they are created near or inside this region suppressing the electron-hole pair recombination^1^. As a result the AQY of Au-ZnO NRs in the UV region was always found to be higher than the bare ZnO NRs, indicating improved photocatalytic activity of the ZnO NRs in the UV region in the presence of the Au NPs, which can be attributed to the enhanced photo-generated charge separation efficiency across the Au-ZnO interface. Additionally, the AQY of ZnO based photocatalysts for MB photocatalytic degradation will also be affected by the formation of leucomethylene blue (LMB) as a byproduct of MB reduction if the process is continued for long time. It has been reported previously that LMB molecules has a tendency to adsorb to the ZnO surface (poisoning the surface), lowering the photocatalytic activity of the ZnO nanostructures after few cycles or with extended irradiation^29^. However, in the present study, such effects will not be significant since AQY was estimated with 30 minutes of monochromatic light excitation, within which LMB production is limited.

In the visible region (λ > 400 nm), the bare ZnO NRs showed slight photocatalytic activity near the beginning of the visible region mainly due to the surface defects present in the NRs^30^, demonstrating an AQY of ~5% at 400 nm and showed almost no activity beyond 450 nm. On the other hand, the Au-ZnO NRs exhibited significantly higher photocatalytic activity in the visible light region, showing remarkably high AQY compared to the bare ZnO NRs. Near the SPR absorption peak of Au NPs at 525 nm, the Au-ZnO NRs demonstrated almost 6 times higher AQY than that of bare ZnO NRs. Under the visible light irradiation, the Au NPs act as light sensitizers injecting photo-generated electrons to the conduction band of ZnO NRs, which has been reported by several researchers showing higher photocatalytic activity upon visible light excitation^5^,^31^. However, it has been reported recently that the quantum efficiency of this injection process in Au-ZnO system is typically very low (in the range of 1–3%)^32^, and therefore its contribution to the overall AQY observed here is actually limited.

In order to probe the high AQY of the Au-ZnO NRs, and hence the plasmonic enhancement of the photocatalytic activity of Au-ZnO NRs in the visible region, we have further investigated the effect of localized plasmonic heat generated by Au NPs upon photo excitation near SPR. At resonance, the oscillating surface plasmons of Au NPs dissipate the energy by two competitive pathways: radiative decay and non-radiative decay. The radiative decay releases energy by the emission of a photon. The efficiency of such emission in Au NPs has been shown to be rather low, with a luminescence quantum yield in the order of 10^−6^ 33. With such a low yield, it is reasonable to exclude the radiative transfer as a major contributor to the enhanced plasmonic photocatalysis. The non-radiative decay, on the other hand, undergoes cascaded steps of electron-electron relaxation, electron-phonon relaxation and finally phonon-phonon relaxation^34^,^35^. The time constants of such non-radiative relaxations can vary from sub-picosecond scale to several picoseconds^36^, and collectively it causes increase in temperature to the surrounding of the metal NPs. Depending on the nature of the metal NPs, local environment and incident light power, the temperature near the surface of the metal NP can rise up to several hundred °C^17^,^18^,^35^,^37^. Such localized heating is beneficial for photocatalytic reactions as reactions can occur at a faster rate at higher temperatures.

**Figure 3:**
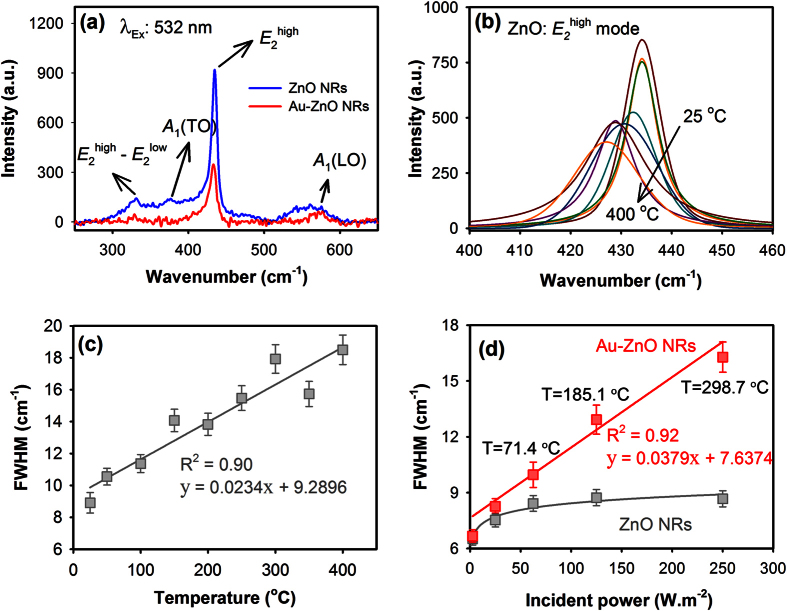
(**a**) Typical Raman spectra of bare ZnO and Au-ZnO NRs measured at room temperature using 532 nm laser excitation. The height and width (FWHM) variations of the *E*_2_^high^ mode of ZnO measured *in-situ* with increasing temperatures are shown in (**b**) and the linear relationship of the FWHM with increasing temperatures is shown in (**c**). The relationship of FWHM of *E*_2_^high^ mode of ZnO in the presence and absence of Au NPs with different laser (532 nm) incident powers is shown in (**d**), where T represents the temperature at the Au-ZnO NRs surface estimated from the corresponding FWHM values.

To estimate the localized heat that is generated by the photo-excited Au NPs, we then carried out Raman spectroscopy and initially followed the phonon spectra of the ZnO NRs measured *in-situ* at different temperatures. Figure 3(a) shows typical Raman spectra of bare ZnO and Au-ZnO NRs measured at room temperature using 532 nm laser excitation. The Raman bands at 331, 376 and 574 cm^‒1^ correspond to the *E*_2_^high^ ‒ *E*_2_^low^, *A*_1_ (TO) and *A*_1_ (LO) phonon modes of ZnO respectively, whereas the strong Raman band observed at 435 cm^‒1^ represents the *E*_2_^high^ mode, which is known to shift and broaden with increasing temperature^38^,^39^. In case of the Au-ZnO NRs, no plasmon enhancement in the phonon modes of ZnO was observed. The intensities of all the phonon modes from ZnO were observed to reduce in the presence of Au NPs, indicating passivation of ZnO surface states upon deposition of Au NPs. We have then followed the FWHM of the *E*_2_^high^ mode of bare ZnO NRs by measuring the Raman signal from ZnO NRs *in-situ* at different temperatures. Figure 3(b) shows the variations in the *E*_2_^high^ mode of bare ZnO NRs measured *in-situ* with increasing temperatures. A linear relationship was observed between the FWHM of the *E*_2_^high^ mode and temperature, as shown in Fig. 3(c), which was then used as a calibration curve to quantify the photo-induced localized heating in Au-ZnO NRs.

The Raman spectra of bare ZnO and Au-ZnO NRs were then collected at room temperature with different incident power density of the 532 nm laser, and the FWHM of the *E*_2_^high^ mode was followed in both the cases as shown in Fig. 3(d). When laser intensity was varied from 2.5–25 W.m^‒2^, the width of the ZnO *E*_2_^high^ mode was observed to increase slightly indicating possible laser induced heating of ZnO surface. However, beyond 25 W.m^‒2^ no significant variations in the width of the ZnO *E*_2_^high^ mode were detected, suggesting that there is no considerable effect from the incident laser intensities on the FWHM of the *E*_2_^high^ mode of ZnO. On the other hand, in the presence of Au NPs, the width of *E*_2_^high^ mode showed linear dependency with increasing laser powers; clearly indicating SPR-induced plasmonic heating in the Au-ZnO NRs. Under the 532 nm laser excitation, the localized heat at the Au-ZnO NRs surface was found to reach close to 300 °C at 250 W.m^‒2^ laser powers, which was obtained by comparing the FWHM of the *E*_2_^high^ mode in Au-ZnO NRs at certain incident laser power with the FWHM value in the calibration curve shown in Fig. 3(c).

**Figure 4:**
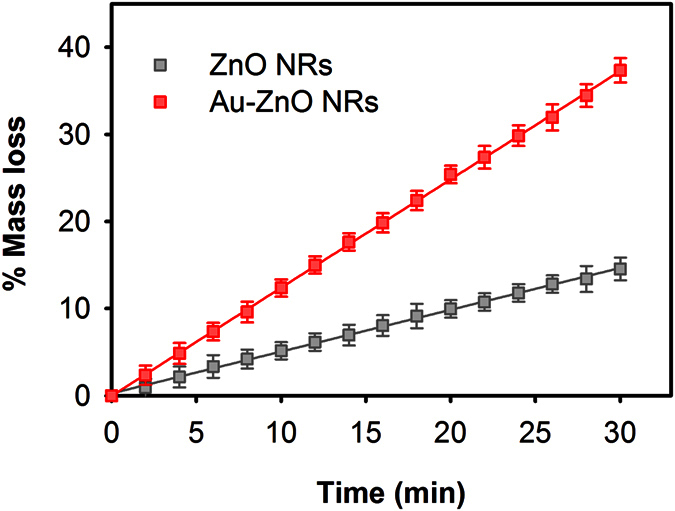
Evaporation kinetics of water from bare ZnO and Au-ZnO NRs surface under 532 nm laser excitations (incident power: 100 W.m^‒2^) as a function of relative mass loss with respect to the laser illumination time. A 20 μl water droplet was dropped on the substrates and the change in mass was monitored using an analytical balance.

The generation of plasmonic heating at the Au-ZnO NRs surface was further investigated by studying the evaporation rate of water from the surface of bare ZnO and Au-ZnO NRs under 532 nm laser excitation with incident power 100 W.m^‒2^, which represents temperature about 90 °C at the Au-ZnO NRs surface obtained from the previous figure. It has been reported recently that under resonant laser excitation a nanometer scale envelope of vapor forms at the Au NPs surface present in a liquid medium due to the localized heating, which grows with time and finally escapes from the NPs surface making the liquid evaporation process faster in the presence of plasmonic Au NPs^40^. To study the water evaporation process, a 20 μl water droplet was dropped on bare ZnO and Au-ZnO NRs surface separately placed on top of a balance, and the evaporation of the water droplet with time was monitored under the laser excitation by recording the weight shown in the balance. Figure 4 shows the evaporation kinetics of water from bare ZnO and Au-ZnO NRs surface under 532 nm laser excitations as a function of weight loss with respect to the laser illumination time. When bare ZnO NRs was used, water was observed to evaporate at an average rate close to 9.89 × 10^‒2^ μl.min^‒1^, while the average evaporation rate was increased by one order (~2.49 × 10^‒1^ μl.min^‒1^) in the presence of the Au NPs on the surface of the ZnO NRs, clearly indicating the generation of localized heat at the surface of the Au-ZnO NRs under the laser excitation.

**Figure 5:**
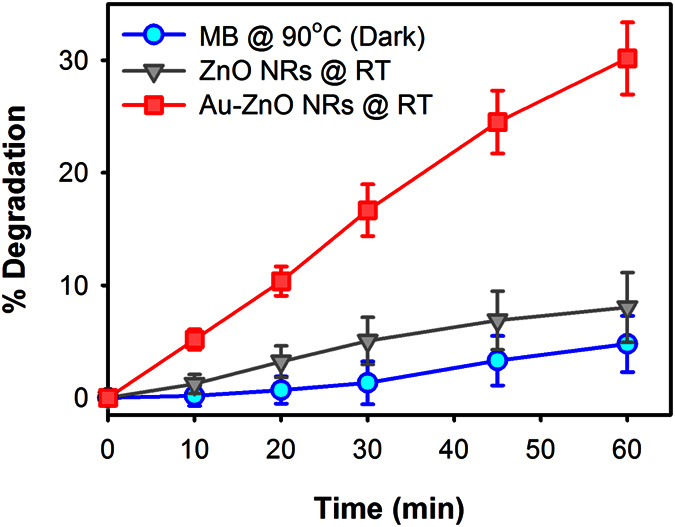
Photocatalytic degradation of MB using bare ZnO and Au-ZnO NRs under 532 nm continuous laser excitation with incident power adjusted to 100 W m^‒2^. MB thermal decomposition at 90 °C in dark for 1 hour (circle) is also shown.

To demonstrate the effect of localized heating directly on plasmonic photocatalysis, we have then carried out MB photocatalysis using the 532 nm laser with incident power adjusted to 100 W.m^‒2^ to obtain localized heat near 90 °C. Figure 5 shows the % degradation of MB under different conditions with 532 nm laser excitation. At 532 nm wavelength, bare ZnO did not show much photocatalytic activity due to its low absorption coefficient at 532 nm. However, in the presence of Au NPs surface plasmon absorption occurs near the 532 nm wavelength resulting injection of electrons from the Au NPs to the conduction band of ZnO NRs and thus creating more e-h pair to actively participate in the photocatalytic reactions. Although the quantum yield of the injection process is low, its contribution in the photocatalytic reactions is enhanced by the localized plasmonic heating rising the temperature at the surface of the Au-ZnO NRs up to about 90 °C, which increases the interaction between the photo-generated charges and MB molecules competing more efficiently with the e-h recombination. As a result compared to the bare ZnO NRs, the Au-ZnO NRs showed almost 3.75 times higher degradation of MB in 1 hour of laser excitation. The thermal decomposition of MB solution at 90 °C in dark was also investigated to verify the effect of heat on the decomposition of MB molecules, which showed less than 5% reduction after 1 hour of heating suggesting relatively less effect of the externally applied heat on the decomposition of MB molecules (thermolysis) and hence, indicating that photocatalysis is the predominant process for the reduction of MB in the presence of Au-ZnO NRs irradiated with visible light.

**Figure 6:**
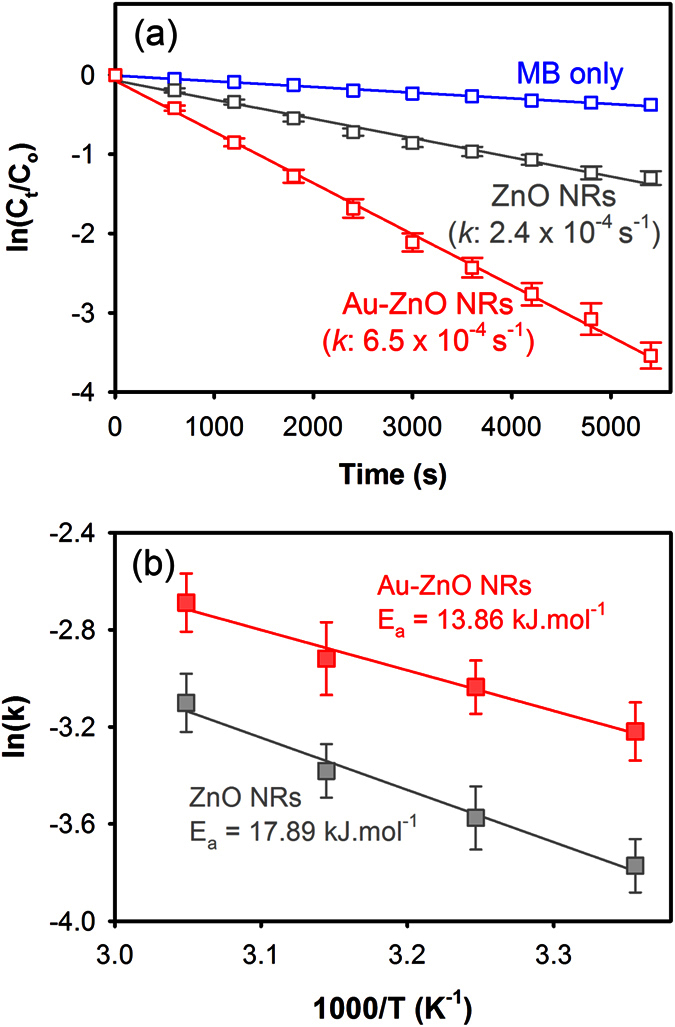
(**a**) Photocatalytic degradation kinetics of MB with bare ZnO and Au-ZnO NRs under solar light (AM 1.5 G) and (**b**) Arrhenius plot showing the activation energy (E_a_) for the photocatalytic degradation of MB under solar light in the presence of bare ZnO and Au-ZnO NRs.

The plasmonic photocatalysis of Au-ZnO NRs under full solar spectrum was then studied and compared with bare ZnO NRs using AM 1.5 G simulated solar light illumination. Figure 6(a) shows the photocatalytic degradation kinetics of MB under simulated solar light with different photocatalysts. In the absence of any photocatalysts, MB degradation was observed to occur very slowly which is purely due to the photolysis of MB molecules under the simulated solar light. In the presence of the bare ZnO NRs, MB molecules were photocatalytically degraded showing a first order relationship with an average rate constant equal to 2.4 × 10^‒4^ s^‒1^. Although bare ZnO NRs do not show significant visible light absorption, its visible light photocatalytic activity is attributed to the surface defects present in the NRs^30^, which allows sub-bandgap absorption in the NRs. The photocatalytic degradation of MB was observed to further enhance in the presence of Au NPs, showing average rate constant equal to 6.5 × 10^‒4^ s^‒1^, which is more than 2.5 times higher than the average rate constant obtained for bare ZnO NRs.

It should be noted here that the enhanced visible light photocatalytic activity of Au-ZnO NRs observed here is not only attributed to the higher visible light absorption by the Au NPs and improved photo-generated charge separation across the Au-ZnO interface, but also to the localized plasmonic heating generated by the Au NPs under resonance which allows improved interactions between the photo-generated charges and the MB molecules despite the limitations from the lower quantum yield of electron injection from excited Au NPs to ZnO NRs. Additionally, the exact amount of localized heat at the surface of the Au-ZnO NRs generated under the full solar spectrum is difficult to determine as the exact incident power at the resonance wavelength cannot be estimated. However, in terms of photocatalytic reactions a small rise in the photocatalyst surface temperature can have significant effect on the photocatalytic activity of the catalyst, which is also contributed by the other plasmonic effects from the Au NPs. In order to verify this we have estimated the activation energy (E_a_) for the photocatalytic degradation of MB under solar light in the presence of bare ZnO and Au-ZnO NRs. Figure 6(b) shows the Arrhenius plot used for the estimation of E_a_, which was obtained by conducting photocatalytic degradation of MB at varying temperatures in the presence of bare ZnO and Au-ZnO NRs. The temperatures for these experiments were controlled by using an oil bath, for which details are given in the Methods section. In the case of the bare ZnO NRs, the E_a_ was found to be about 17.89 (±2.24) kJ.mol^‒1^, which was then reduced by almost 22.5% to 13.86 (±1.67) kJ.mol^‒1^ upon incorporation of Au NPs on the ZnO NRs clearly indicating the reduction in activation energy due to the localized plasmonic heating and other plasmonic effects allowing the photocatalytic reactions to occur at a faster rate.

In summary, plasmonic Au NPs were deposited on the surface of vertically aligned ZnO nanorods and the effect of localized plasmonic heating from the Au NPs under the resonance excitation on the visible light photocatalytic activity of Au-ZnO NRs was explored in details. Using Raman spectroscopy the surface temperature of the Au-ZnO NRs was experimentally determined by following the phonon modes of ZnO, which revealed that the surface temperature of the Au-ZnO NRs under the resonant excitation of Au NPs can reach as high as ~300 °C depending on the incident power of the laser. Rise of the temperature at the Au-ZnO photocatalysts surface was found to be beneficial towards the improvement of the AQY of Au-ZnO NRs resulting in almost 6 times higher AQY compared to the bare ZnO NRs. As a result of the localized plasmonic heating and other plasmonic effects from Au NPs the activation energy required for the photocatalytic degradation of MB was reduced by 22.5% in the presence of the Au NPs, which demonstrated visible light photocatalytic activity almost double than the bare ZnO NRs under the solar light irradiation. The results from this study showed that localized plasmonic heating has a crucial role in defining the efficacy of the Au-ZnO based plasmonic photocatalysts apart from their higher visible light harvesting and improved charge separation across the metal-semiconductor interface and thus provide detailed insight on the visible light driven plasmonic photocatalysis process.

## Methods

### Synthesis of Au-ZnO NRs

In order to prepare the Au-ZnO NRs, initially ZnO NRs were grown on glass substrates by a microwave assisted hydrothermal method^30^. First the glass substrates were cleaned ultrasonically in soap water, acetone, ethanol and DI water and dried in an oven. A ZnO seed layer was then deposited on the cleaned glass substrates by spraying 10 ml of 5 mM aqueous solution of zinc acetate at 350 °C. The seeded substrates were then immersed in a beaker containing an aqueous solution of 20 mM zinc nitrate and 20 mM hexamethylenetetramine and the growth of the NRs was then carried out in a commercial microwave oven with microwave output power of 180 W. The growth process is consists of two steps: 40 minutes of growth under microwave irradiation and 20 minutes of cooling in ambient. The ZnO NRs growth was continued for 5 hours, where the growth solution was replenished in every 1 hour in order to maintain a constant growth rate of the NRs during the hydrothermal process. Finally the substrates were retracted from the chemical bath, rinsed thoroughly with DI water followed by annealing the NRs at 250 °C for 1 hour in ambient in order to remove any organic residues present on the surface of the NRs.

Au NPs were then deposited *in situ* on the surface of the as prepared ZnO NRs by photocatalytically reducing chloroauric acid (HAuCl_4_.3 H_2_O) using UV irradiation. In a typical deposition process, ZnO NRs were immersed in a 0.1 mM aqueous solution of HAuCl_4_.3H_2_O, followed by irradiation with UV light (18 W) for 10 minutes. During the process ZnO NRs absorb the high energy UV light and generate electron–hole (e-h) pairs, which lead to the formation of highly reactive radicals with the subsequent reduction of AuCl_4_^‒^ ions to metallic Au on the surface of the NRs. After the deposition, the Au-ZnO NRs coated glass substrates were rinsed with copious amount of DI water and annealed at 450 °C for 1 hour in ambient.

### Characterization

Au-ZnO NRs were characterized by transmission electron microscopy (TEM; Model: JEOL JEM-2100F operated at 200 kV) and scanning electron microscopy (SEM; Model: JEOL JSM-7600F, operated at 20 kV). Optical absorption spectra of the samples were recorded by using a Perkin Elmer Lambda 25 UV/Vis spectrometer. Raman spectroscopy (XploRA from Horiba) was used to probe the localized plasmonic heating from the Au NPs deposited on the surface of the ZnO NRs with continuous 532 nm laser excitation (laser spot size: 2 μm). A small piece (3 mm × 3 mm) glass substrate coated with ZnO NRs without Au NPs was initially placed on a heating stage (T95-HT from Linkam) comprising of a ceramic heating cell where the sample was in direct contact with the cell and the temperature of the cell was increased from 25 °C to 400 °C at a rate of 5 °C/min controlled by the Linkam T95-HT controller. *In-situ* Raman spectra were collected at every 50 °C temperature interval from 200–700 cm^‒1^ using a TE cooled CCD camera (Syncerity) attached to the monochromator of a spectrometer with 1800 gr/mm grating and spectral resolution better than 2 cm^‒1^. An integration time of 20 seconds was fixed for all Raman measurements. In a similar way Au-ZnO NRs samples were placed in the heating cell while the temperature was maintained at 25 °C and the Raman spectra were recorded with continuous 532 nm laser excitation at different incident laser powers ranging from 2.5–250 W.m^‒2^ in order to follow the phonon modes of ZnO at different laser excitation powers. The incident laser power was controlled by using a motorized density filter inbuilt to the Raman system.

### Photocatalytic tests

Photocatalysis tests were conducted using an aqueous solution of methylene blue (MB) as a test contaminant. Simulated solar irradiation (AM 1.5G, incident power: 1000 W.m^‒2^) from a solar simulator (Sciencetech, Model: SS1.6 kW) fitted with IR filter was used as the visible light source. A 10 μM solution of MB was prepared in DI water and placed in poly(- methyl methacrylate) (PMMA) cuvettes. A glass substrate containing the bare ZnO or Au-ZnO NRs (3 cm × 1 cm) were then placed inside the cuvette with the catalyst surface facing the light source. As control, a bare glass substrate of similar size was placed in a cuvette containing the MB solution. Optical absorption spectra of the MB solution were then recorded after different light exposure durations in order to monitor the rate of photocatalytic degradation of the test contaminant. Prior to the photocatalytic degradation, the samples were kept in dark for 1 hour in the MB solution to reach adsorption equilibrium. The photocatalytic degradation of MB was estimated from the reduction in absorption intensity of MB at a fixed wavelength λ_max_ = 664 nm. All the degradation curves were then plotted using the integral representation form of a first order reaction, which can be mathematically given as equation (1):


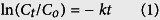


where, *C*_*t*_ is the concentration of MB at time *t*, *C*_*o*_ is the initial concentration of MB at time 0 and *k* is the first order rate constant. The values of *k* were then estimated from the slope of the linear fitting curve of equation 1 and represented in s^−1^.

In order to study the photocatalytic activity of bare ZnO and Au-ZnO NRs under resonance excitation of Au NPs, photocatalytic degradation of MB was carried out in the similar way with a 532 nm continuous laser (incident power: 100 W.m^‒2^) as the light source instead of the simulated solar light.

### Estimation of Apparent Quantum Yield (AQY)

AQY of bare ZnO and Au-ZnO NRs for the photocatalytic degradation of MB was calculated for different incident monochromatic wavelengths ranging from 300–600 nm and the photocatalytic degradation of MB at each wavelength was conducted for 30 minutes. In order to excite the photocatalysts with specific wavelengths we have used a monochromator from Perkin Elmer fitted with a high energy pulsed xenon source for excitation. An inbuilt holographic grating was used to obtain the specific wavelengths ensuring very low stray light radiation.

Quantum yield (QY), which is widely used to evaluate the performance of photocatalysts, is defined by the following equation:
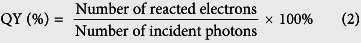


However, it is difficult to directly determine the “number of reacted electrons” via experimental methods. In order to estimate that, first we have measured the number of MB molecules degraded in 30 minutes from the recorded optical absorption at 664 nm. It has been reported earlier that photocatalytic degradation of MB occurs predominantly through reduction process resulting in the formation of semi reduced MB (MB radical), which upon subsequent reduction forms colorless leucomethylene blue (LMB)^41^. Therefore, the complete reduction process of MB requires two electrons to produce LMB. With this assumption, the AQY was estimated as:


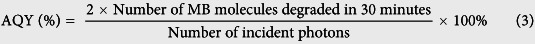


The number of MB molecules degraded in 30 minutes and the number of incident photons was then estimated by using equations (4) and (5) respectively.


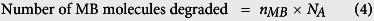



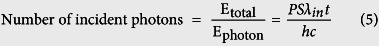


where, *n*_*MB*_ (mol) is the amount of MB degraded over the duration *t* of the incident light exposure (determined from optical absorption spectra), *N*_*A*_ (mol^‒1^) is Avogadro’s constant, *P* (W.m^‒2^) is the power density of the incident monochromatic light, *S* (m^2^) is the irradiation area, *t* (s) is the duration of the incident light exposure which is 30 minutes (1800 s) in this case and *λ*_*in*_ (m) is the wavelength of the incident monochromatic light. The incident power density (*P*) at different excitation wavelengths were measured by using a pyranometer from Kipp & Zonen (CMP 3), by keeping the distance between the monochromator and the pyranometer same as the distance between the monochromator and the photocatalyst substrates during the photocatalysis process.

The AQY of each system was then estimated by combining equations (4) and (5), which is given as:


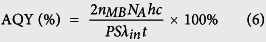


### Estimation of activation energy (E_a_)

The activation energy (E_a_) for photocatalytic degradation of MB with bare ZnO and Au-ZnO NRs as photocatalysts under solar light was estimated by using Arrhenius plot [ln(k) vs.1/T], where the rate of photocatalytic degradation (*k*) of MB at different temperatures (T) was calculated by conducting photocatalytic tests at different MB solution temperatures. The temperature of the MB solutions was controlled by using an oil bath, where the cuvette containing MB solutions were dipped into the oil bath placed on a hot plate and kept in dark for sufficient time until the temperature of the MB solution reached to the desired level, which was continuously monitored using a thermocouple connected to a temperature reader. Photocatalytic tests under simulated solar light were then conducted while maintaining the temperature using the oil bath following the same procedure used for other photocatalytic tests in the work. The E_a_ value was then estimated from the slope (E_a_/k_B_) of the Arrhenius plot, where k_B_ represents the Boltzmann constant.

## Additional Information

**How to cite this article**: Bora, T. *et al.* Importance of Plasmonic Heating on Visible Light Driven Photocatalysis of Gold Nanoparticle Decorated Zinc Oxide Nanorods. *Sci. Rep.* **6**, 26913; doi: 10.1038/srep26913 (2016).

## Supplementary Material

Supplementary Information

## References

[b1] Zhang X., Chen Y. L., Liu R. S. & Tsai D. P. Plasmonic photocatalysis. Rep. Prog. Phys. 76, 046401 (2013). 23455654 10.1088/0034-4885/76/4/046401

[b2] Wang P., Huang B., Dai Y. & Whangbo M.-H. Plasmonic photocatalysts: harvesting visible light with noble metal nanoparticles. Phys. Chem. Chem. Phys. 14, 9813–9825 (2012). 22710311 10.1039/c2cp40823f

[b3] Pincella F., Isozaki K. & Miki K. A visible light-driven plasmonic photocatalyst. Light Sci. Appl. 3, e133 (2014).

[b4] Zhang X., Ke X., Du A. & Zhu H. Plasmonic nanostructures to enhance catalytic performance of zeolites under visible light. Sci. Rep. 4, 3805 (2014). 24448225 10.1038/srep03805 PMC3898204

[b5] Bora T., Myint M. T. Z., Al-Harthi S. H. & Dutta J. Role of surface defects on visible light enabled plasmonic photocatalysis in Au-ZnO nanocatalysts. RSC Adv. 5, 96670 (2015).

[b6] Chen X., Zhu H.-Y., Zhao J.-C., Zheng Z.-F. & Gao X.-P. Visible-light-driven oxidation of organic contaminants in air with gold nanoparticle catalysts on oxide supports. Angew. Chem. Int. Ed. 120, 5433–5436 (2008). 10.1002/anie.200800602 18548470

[b7] Zhang P. *et al.* Core/shell nanofibers of TiO_2_@carbon embedded by Ag nanoparticles with enhanced visible photocatalytic activity. J.Mater. Chem. 21, 17746–17753 (2011).

[b8] Naya S.-I., Inoue A. & Tada H. Self-assembled heterosupramolecular visible light photocatalyst consisting of gold nanoparticle-loaded titanium(IV) dioxide and surfactant. J. Am. Chem. Soc. 132, 6292–6293 (2010). 20397694 10.1021/ja101711j

[b9] Saoud K. *et al.* Synthesis of supported silver nano-spheres on zinc oxide nanorods for visible light photocatalytic applications. Mater. Res. Bull. 63, 134–140 (2015).

[b10] Hao Q. *et al.* Aluminum plasmonic photocatalysis. Sci. Rep. 5, 15288 (2015). 26497411 10.1038/srep15288 PMC4620498

[b11] Ullah R. & Dutta J. Photocatalytic degradation of organic dyes with manganese-doped ZnO nanoparticles. J. Hazard. Mater. 156, 194–200 (2008). 18221834 10.1016/j.jhazmat.2007.12.033

[b12] Kanade K. G. *et al.* Self-assembled aligned Cu doped ZnO nanoparticles for photocatalytic hydrogen production under visible light irradiation. Mater. Chem. Phys. 102, 98–104 (2007).

[b13] Moon J., Yun C. Y., Chung K. W., Kang M. S. & Yi J. Photocatalytic activation of TiO_2_ under visible light using Acid Red 44. Catal. Today 87, 77–86 (2003).

[b14] Sarkar S. *et al.* Hematoporphyrin–ZnO nanohybrids: twin applications in efficient visible-light photocatalysis and dye-sensitized solar cells. ACS Appl. Mater. Interfaces 4, 7027–7035 (2012). 23186038 10.1021/am302288m

[b15] Ma X.-C., Dai Y., Yu L. & Huang B.-B. Energy transfer in plasmonic photocatalytic composites. Light Sci. Appl. 5, e16017 (2016). 30167139 10.1038/lsa.2016.17 PMC6062428

[b16] Adleman J. R., Boyd D. A., Goodwin D. G. & Psaltis D. Heterogenous catalysis mediated by plasmon heating. Nano Lett. 9, 4417–4423 (2009). 19908825 10.1021/nl902711n

[b17] Govorov A. O. & Richardson H. H. Generating heat with metal nanoparticles. Nano Today 2, 30–38 (2007).

[b18] Baffou G., Quidant R. & Girard C. Heat generation in plasmonic nanostructures: Influence of morphology. Appl. Phys. Lett. 94, 153109 (2009).

[b19] Govorov A. O. *et al.* Gold nanoparticle ensembles as heaters and actuators: melting and collective plasmon resonances. Nanoscale Res. Lett. 1, 84–90 (2006).

[b20] Baffou G., Kreuzer M. P., Kulzer F. & Quidant R. Temperature mapping near plasmonic nanostructures using fluorescence polarization anisotropy. Opt. Express 17, 3291–3298 (2009). 19259165 10.1364/oe.17.003291

[b21] Carlson M. T., Khan A. & Richardson H. H. Local temperature determination of optically excited nanoparticles and nanodots. Nano Lett. 11, 1061–1069 (2011). 21306114 10.1021/nl103938u

[b22] Qiu J. *et al.* Surface plasmon mediated chemical solution deposition of gold nanoparticles on a nanostructured silver surface at room temperature. J. Am. Chem. Soc. 135, 38–41 (2013). 23241020 10.1021/ja309392x

[b23] Richardson H. H. *et al.* Thermooptical properties of gold nanoparticles embedded in ice: Characterization of heat generation and melting. Nano Lett. 6, 783–788 (2006). 16608284 10.1021/nl060105l

[b24] Lee J., Govorov A. O. & Kotov N. A. Nanoparticle assemblies with molecular springs: A nanoscale thermometer. Angew. Chem. Int. Ed. 44, 7439–7442 (2005). 10.1002/anie.200501264 16231378

[b25] Wang C. *et al.* Visible light plasmonic heating of Au-ZnO for the catalytic reduction of CO2. Nanoscale 5, 6968–6974 (2013). 23794025 10.1039/c3nr02001k

[b26] Huang X. & El-Sayed M. A. Gold nanoparticles: Optical properties and implementations in cancer diagnosis and photothermal therapy. J. Adv. Res. 1, 13–28 (2010).

[b27] Jain P. K., Lee K. S., El-Sayed I. H. & El-Sayed M. A. Calculated absorption and scattering properties of gold nanoparticles of different size, shape, and composition: Applications in biological imaging and biomedicine. J. Phys. Chem. B 110, 7238–7248 (2006). 16599493 10.1021/jp057170o

[b28] Link S. & El-Sayed M. A. Size and temperature dependence of the plasmon absorption of colloidal gold nanoparticles. J. Phys. Chem. B 103, 4212–4217 (1999).

[b29] Baruah S. *et al.* Photoreactivity of ZnO nanoparticles in visible light: Effect of surface states on electron transfer reaction. J. Appl. Phys. 105, 0743081–0743086 (2009).

[b30] Baruah S., Mahmood M. A., Myint M. T. Z., Bora T. & Dutta J. Enhanced visible light photocatalysis through fast crystallization of zinc oxide nanorods. Beilstein J. Nanotechnol. 1, 14–20 (2010). 21977391 10.3762/bjnano.1.3 PMC3045919

[b31] Tian Y. & Tatsuma T. Mechanisms and applications of plasmon-induced charge separation at TiO_2_ films loaded with gold nanoparticles. J. Am. Chem. Soc. 127, 7632–7637 (2005). 15898815 10.1021/ja042192u

[b32] Pescaglini A. *et al.* Hot-electron injection in Au nanorod–ZnO nanowire hybrid device for near-infrared photodetection. Nano Lett. 14, 6202–6209 (2014). 25313827 10.1021/nl5024854

[b33] Dulkeith E. *et al.* Plasmon emission in photoexcited gold nanoparticles. Phys. Rev. B 70, 205424 (2004).

[b34] Link S. & El-Sayed M. A. Spectral properties and relaxation dynamics of surface plasmon electronic oscillations in gold and silver nanodots and nanorods. J. Phys. Chem. B 103, 8410–8426 (1999).

[b35] Link S. & El-Sayed M. A. Shape and size dependence of radiative, non-radiative and photothermal properties of gold nanocrystals. Int. Rev. Phys. Chem. 19, 409–453 (2000).

[b36] Sun C. K., Vallée F., Acioli L. H., Ippen E. P. & Fujimoto J. G. Femtosecond-tunable measurement of electron thermalization in gold. Phys. Rev. B 50, 15337–15348 (1994). 10.1103/physrevb.50.15337 9975886

[b37] Baffou G., Girard C. & Quidant R. Mapping heat origin in plasmonic structures. Phys. Rev. Lett. 104, 136805 (2010). 20481904 10.1103/PhysRevLett.104.136805

[b38] Cuscó R. *et al.* Temperature dependence of Raman scattering in ZnO. Phys. Rev. B 75, 165202 (2007).

[b39] Yadav H. K., Katiyar R. S. & Gupta V. Temperature dependent dynamics of ZnO nanoparticles probed by Raman scattering: A big divergence in the functional areas of nanoparticles and bulk materials. Appl. Phys. Lett. 100, 051906 (2012).

[b40] Fang Z. *et al.* Evolution of light-induced vapor generation at a liquid-immersed metallic nanoparticle. Nano Lett. 13, 1736–1742 (2013). 23517407 10.1021/nl4003238 PMC3888228

[b41] Impert O. *et al.* Kinetics and mechanism of a fast leuco-Methylene Blue oxidation by copper(ii)-halide species in acidic aqueous media. Dalton Trans. 3, 348–353 (2003).

