# Supplementary material for: Importance of Plasmonic Heating on Visible Light Driven Photocatalysis of Gold Nanoparticle Decorated Zinc Oxide Nanorods
Source: Sci Rep. 2016 May 31;6:26913. doi: 10.1038/srep26913 (PMC4886257; doi:10.1038/srep26913)
Supplement: Supplementary Information [file srep26913-s1.pdf]

SUPPLEMENTARY INFORMATION

Importance of Plasmonic Heating on Visible Light Driven Photocatalysis of Gold Nanoparticle Decorated Zinc Oxide Nanorods

Tanujjal Bora[1*], *David Zoepfl*[2] and *Joydeep Dutta*[1, 3*]

[1]*Chair in Nanotechnology, Sultan Qaboos University, PO Box 17, Al Khoud–123, Oman*

[2]*Institute for Quantum Optics and Quantum Information, Austrian Academy of Sciences, Technikerstraße 21a, A-6020 Innsbruck, Austria*

[3] *Functional Materials Division, Materials and Nano Physics Department, ICT School, KTH Royal Institute of Technology, SE-164 40, Kista, Stockholm, Sweden*

***Corresponding author:** tanujjal@squ.edu.om, joydeep@kth.se

Energy Dispersive X-ray (EDX) Analysis of Au-ZnO NRs

The amount of Au loading on ZnO NRs was varied by changing the deposition time of Au NPs from 5 to 10 minutes. The Au loading was then determined by using EDX analysis. Figure S1 shows a typical EDX spectrum of Au-ZnO NRs where Au NPs were deposited by photocatalytic reduction of chloroauric acid ($HAuCl_4.3H_2O$) for 10 minutes. The variation in the Au loading, as a function of deposition time, is also shown in the inset. With increasing deposition time from 5 to 10 minutes, the Au concentration was also observed to increase almost linearly from 1.5 ± 0.1 to 2.6 ± 0.2 wt. % respectively.

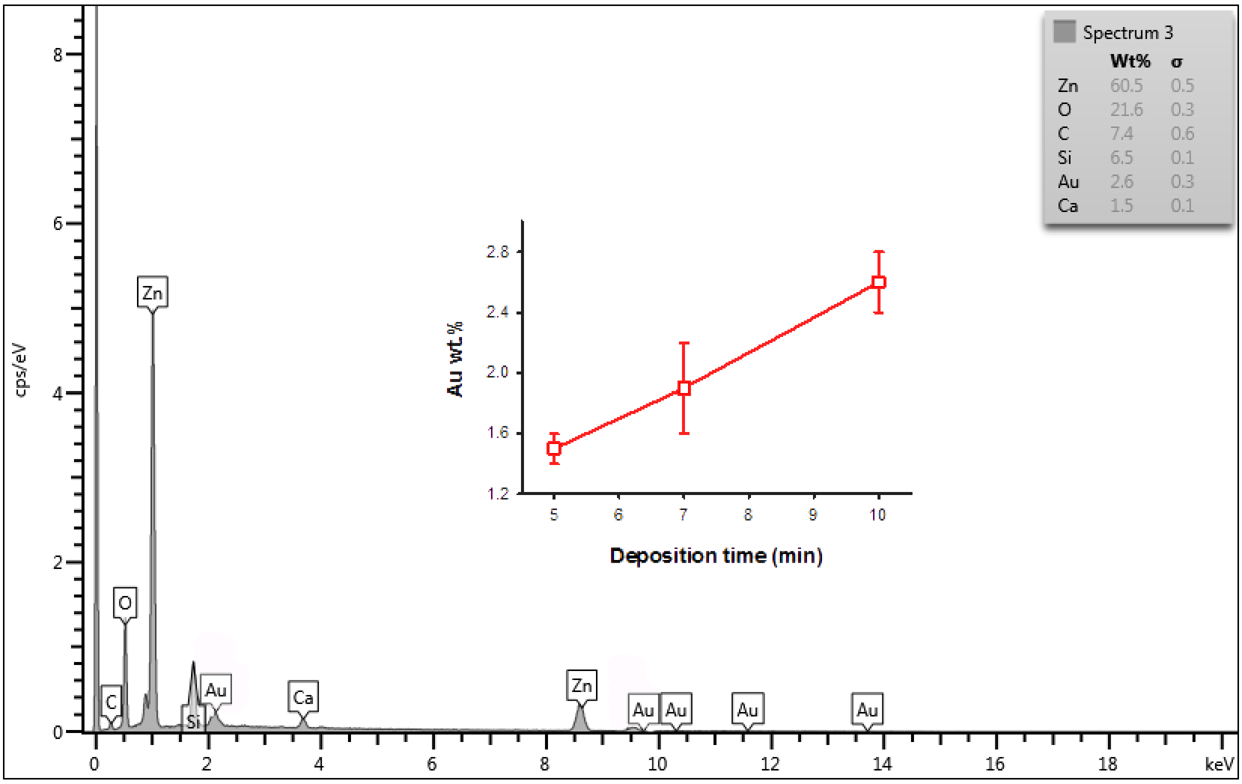

Figure S1. A typical EDX spectrum of Au-ZnO NRs, where Au NPs were deposited on the surface of ZnO NRs by photocatalytic reduction of chloroauric acid ($HAuCl_4.3H_2O$) for 10 minutes under UV irradiation. Inset shows the variations in Au loading with different deposition time obtained from EDX analysis in the Au-ZnO NRs.

Synthesis of Colloidal Au NPs

Citrate capped colloidal Au NPs were synthesized in aqueous medium using a modified Turkevitch process[1]. In a typical synthesis process 2 ml of 5 mM gold chloride hydrate ($HAuCl_4.H_2O$) aqueous solution was added to 50 ml of deionized (DI) water in a conical flask and the mixture was heated to boil by placing it on a hot plate at 120 °C. Upon boiling, 2.8 ml of 25 mM trisodium citrate (TSC, $Na_3C_6H_5O_7.2H_2O$) aqueous solution was added drop wise to it under vigorous stirring. TSC being a mild reducing agent reduces gold chloride solution that leads the colorless solution to turn wine red, indicating the formation of Au NPs. Once the color of the solution reached to wine red and remained unchanged for about a

minute, the conical flask was dipped immediately in an ice cold water bath in order to quench the reaction or stop further growth of the Au NPs. The as obtained colloidal Au NPs were then stored in refrigerator for further use.

Figure S2 shows a typical TEM micrograph of the as prepared Au NPs along with the size distribution of the NPs. All Au NPs exhibited almost spherical shape with maximum size around 20 nm. The optical absorption of these Au NPs showed SPR peak around 525 nm, which is shown in Figure 1(e) of the main manuscript.

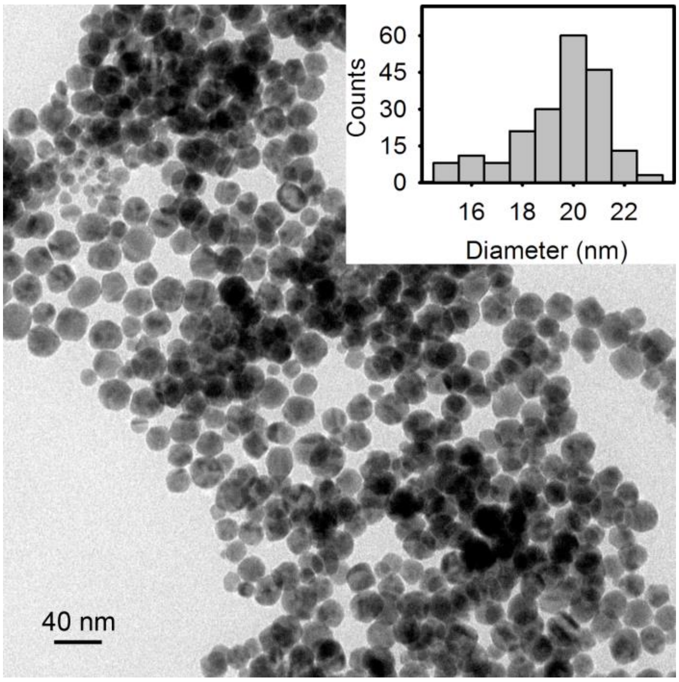

Figure S2. TEM micrograph of colloidal Au NPs showing the size distribution of the NPs (inset).

References

1. Turkevich, J. Colloidal Gold. Part I. *Gold Bulletin* **18**, 86-91 (1985).
